# The validation of an educational database for children with profound intellectual disabilities

**DOI:** 10.4102/ajod.v5i1.237

**Published:** 2016-09-23

**Authors:** Karlien Spangenberg, Lieselotte Corten, Winnie van Rensburg, Elizma Kilian, Judith McKenzie, Hein Vorster, Jennifer Jelsma

**Affiliations:** 1 Children with Severe and Profound Intellectual Disabilities Inclusive Education Outreach Team, Western Cape Department of Education, South Africa; 2 Department of Health and Rehabilitation Sciences, University of Cape Town, South Africa

## Abstract

**Background:**

The Western Cape Forum for Intellectual Disability took the South African Government to court in 2010 on its failure to implement the right to education for Children with Severe and Profound Intellectual Disability. Subsequently, multidisciplinary teams were appointed by the Western Cape Education Department to deliver services to the Special Care Centres (SCCs). Initially, minimal information was available on this population.

**Objectives:**

The purpose is to document the process of developing and validating a database for the collection of routine data.

**Method:**

A descriptive analytical study design was used. A sample of convenience was drawn from individuals under the age of 18 years, enrolled in SCCs in the Western Cape. The team who entered and analysed the data reached consensus regarding the utility and feasibility of each item.

**Results:**

Data were collected on 134 children. The omission of certain items from the database was identified. Some information was not reliable or readily available. Of the instruments identified to assess function, the classification systems were found to be reliable and useful, as were the performance scales. The WeeFIM, on the other hand, was lengthy and expensive, and was therefore discarded.

**Discussion and conclusions:**

A list of items to be included was identified. Apart from an individual profile, it can be useful for service planning and monitoring, if incorporated into the central information system used to monitor the performance of all children. Without such inclusion, this most vulnerable population, despite court ruling, will not have their right to education adequately addressed.

## Introduction

The second United Nations (UN) Millennium Development Goals included the achievement of universal primary education (UN) by 2015, which encompasses children with disabilities. Yet for millions of children with physical and intellectual disability this goal may not be close to realisation, despite various UN Declarations affirming their rights to education. Children with more severe and profound disabilities have been especially disadvantaged.

Within the Western Cape (WC), there are estimated to be approximately 3000 children with severe or profound intellectual disability (CSPID) (Kleintjies *et al*. 2006; McKenzie, McConkey & Adnams 2013a). In this study, Kleintjies *et al*. defines profound intellectual disability as having IQ levels below 30. The DSM-5 (American Psychiatric Association 2013) stipulates the following specifiers in terms of the severity of intellectual disability:

The various levels of severity are defined on the basis of adaptive functioning, and not IQ scores, because it is adaptive functioning that determines the level of supports required. Moreover, IQ measures are less valid in the lower end of the IQ range.

Therefore, this population of children has both intellectual and adaptive functioning deficits in the domains of conceptual, social and practical domains. In the practical domain (American Psychiatric Association 2013), it means that:

The individual requires support for all activities of daily living, including meals, dressing, bathing, and elimination. The individual requires supervision at all times. The individual cannot make responsible decisions regarding well-being of self or others.

The prevalence of sensory impairments, visual and hearing, in people with intellectual disabilities is 10 and 40–100 times greater than in the general population (Carvill 2001). Prior to the court action described below, there was little recognition that these children had the right to educational services provided by the National Department of Basic Education, a situation similar to most African countries (McKenzie, McConkey & Adnams 2013b).

The Western Cape Forum for Intellectual Disability, on behalf of its members, took the South African Government to court on the matter of the rights of CSPID in the WC in 2007 (South African Legal Information Institute 2007). The application was successful, and the judgement required the Western Cape Education Department (WCED) to take incremental steps to ensure that such children have affordable access to a basic education of an adequate quality and to report back on the progress made in implementing the judgement. Implementation entailed supporting organisations and centres that provide education and care to CSPID.^1^ As a consequence, four multidisciplinary educational teams were appointed to work with CSPID in special care centres (SCCs) in the WC. Each team consists of a psychologist, a learning support educator, an occupational therapist, a physiotherapist, and a speech and language therapist. The teams supported approximately 35 SCCs in six of the eight education districts in the WC by March 2013.^2^ This amounts to about 1050 children and about 120 care staff at the SCCs.

The four CSPID teams have adopted a phased approach to the roll out of educational inclusion in the SCCs, beginning with relationship building with the centre staff, identifying the children, consulting with parents, formalising the content and scope for programme development and teaching care staff to provide educational stimulation to the children. Goals are set and prioritised for each child. Centre caregiver training is done on site, and this is practical and interactive in nature. The various team members are consulted by a representative involved in developing a national curriculum for profound intellectual disability (PID), which links with the National Curriculum Framework for children birth to four years and the curriculum for severe intellectual disability, under development.

At the commencement of the programme, there was very little information available with regard to the demographics, educational accomplishments or functional limitations of the children enrolled in the programme. As a result, there was no baseline information from which to map the progress of the children and little evidence to monitor the efficacy of the programmes. Literature review on similar projects around the globe did not render satisfactory results. Apparent inconsistencies in the definitions of disability hamper international comparison (Robson & Evans 2003), especially true in special education, in which different systems lead to even more controversy (Robson & Evans 2003).

Every typically developing child has their educational progress monitored by the national Department of Education, through the Central Education Management Information System (CEMIS). There was thus a need to develop a database appropriate for the needs of CSPID. This database should form part of systems for assessing and monitoring changes in children’s physical, social, communication and cognitive competences as well as their health, personal care and emotional well-being. Children attending these SCCs are only the tip of the iceberg of CSPID, as many children are still excluded from any form of education. However, due to the brief and logistical constraints of the multifunctional teams, this study could not include these children.

### Aim of the study

The purpose of this paper is to document the process of developing and validating a database for the collection of routine data for CSPID. This could potentially form part of CEMIS and be administered centrally. It is hoped that the documentation of this process will assist other organisations wishing to develop similar databases.

## Research method and design

An iterative process, including group discussions, training sessions and online discussion, was utilised to reach consensus on suitable items for the database. A descriptive analytical study design was used to pilot the prototype database.

### Participants

The participants included eight to ten members. To objectify the development of the database, the representatives of the four CSPID teams were joined by representatives from within the Department of Health and Rehabilitation Sciences of the University of Cape Town. The participants represented the disciplines of psychology, teaching, occupational therapy, speech and language therapy, and physiotherapy. Information gained through the initial audit of the SCCs and the subsequent findings of the original team were considered in this process.¹ The data collection form was rolled out to 12 centres in 3 of the rural districts by September 2014 to assess all children in these centres, as part of their routine management. The sample for the validation study was drawn from all individuals under the age of 18 years, who were enrolled in 10 of the 12 centres serviced by the rural team, as indicated on registers for September 2014. The exclusion of two of these centres was due to logistical constraints in service delivery at that time. As the purpose was to explore the feasibility and usefulness of the data collection form, the sample was one of convenience and not necessarily representative of all 273 individuals serviced by the rural team by September 2014. The results were then presented to the members of the small task team who reached consensus with regard to which items should be included in the definitive database.

### Instrumentation

The database items were identified through a series of consensus meetings. The multidisciplinary nature of the team contributed to the face validity of the instrument. There was considerable discussion as to which demographic and health condition items should be included, as well as items relating to medical management, provision of assistive devices and therapeutic interventions.

All members were requested to identify standardised instruments that were valid and responsive for the measurement of the different aspects of functioning of each child. The criteria for inclusion were that the instrument should be able to be used by any member of the team, that it should be robust and that it would monitor important functional and educational skills in the target population. The standardised instruments considered included, amongst others, the Gross Motor Function Measure (McDowell 2008), the Alberta Infant Motor Scale (Piper *et al*. 1992), the Bayley Scales of Infant Development (Milne, McDonald, Comino 2012), the Vineland Social Emotional Early Childhood Scale (Van Duijn *et al*. 2009) and the Receptive One Word Vocab Test (Tafiadis *et al*. 2010). However, it was agreed that each of these tests required specialised, discipline specific skills to be used routinely by any member of the team.

The instruments that were ultimately chosen included different classification systems: the Gross Motor Function Classification System (GMFCS) (McDowell 2008), the Manual Ability Classification System (MACS) (Eliasson *et al*. 2006) and the Communication Function Classification System (CFCS) (Hidecker *et al*. 2011). These were included to give a gross measurement of the level of functioning of the children and a summary can be found in Tables 1–3.

**TABLE 1:** Gross motor function classification system.

Level	General heading description
Level I	Walks without limitations
Level II	Walks with limitations
Level III	Walks using a hand-held mobility device
Level IV	Self mobility with limitations; may use powered wheelchair
Level V	Transported in a manual wheelchair

*Source:* GMFCS – E & R © Robert Palisano, Peter Rosenbaum, Doreen Bartlett, Michael Livingstone 2007 *CanChild* Centre for Childhood Disability Research, McMaster University

**TABLE 2:** Manual ability function classification system.

Level	Description
Level I	Handles objects easily and successfully
Level II	Handles most objects but with somewhat reduced quality and/or speed of achievement
Level III	Handles objects with difficulty; needs help to prepare and/or modify activities
Level IV	Handles a limited selection of easily managed objects in adapted situations
Level V	Does not handle objects and has severely limited ability to perform even simple actions

*Source:* Eliasson AC, Krumlinde Sundholm L, Rösblad B, Beckung E, Arner M, Öhrvall AM. The Manual Ability Classification System (MACS) for children with cerebral palsy: scale development and evidence of validity and reliability. Developmental Medicine and Child Neurology 2006 48:549–554

**TABLE 3:** Communication function classification system.

Level	Description
Level I	Effective sender and receiver with unfamiliar and familiar partners
Level II	Effective but slower paced sender and/or receiver with unfamiliar and/or familiar partners
Level III	Effective sender and receiver with familiar partners
Level IV	Inconsistent sender and/or receiver with familiar partners
Level V	Seldom effective sender and receiver even with familiar partners

*Source:* Hidecker, M.J.C., Paneth, N., Rosenbaum, P.L., Kent, R.D., Lillie, J. *et al.* 2011 Developing and validating the Communication Function Classification System (CFCS) for individuals with cerebral palsy, Developmental Medicine and Child Neurology. 53(8), 704–710

In addition, the WeeFIM (Ottenbacher *et al*. 2000), which gathers data on self-care, mobility and cognition, was included to monitor the independent functioning of the children.

The performance scales (P-scales), which are the national curriculum performance attainment targets for children with special education needs in the UK, were also included (Department for Education 2014). The attainment scales are differentiated performance criteria and give an idea of the child’s level of participation to structured activities throughout the day, hence measuring educational-oriented achievement. The P-scales use eight performance levels to illustrate learning. Levels P1 to P3 show the earliest levels of general attainment. Levels P4 to P8 show subject-related attainment, focusing on extending understanding and connecting knowledge. The extracts from the P-Scale level descriptions 2009 in Table 4 will assist in understanding the value and relevance of this tool (Qualifications and Curriculum Authority 2009).

**TABLE 4:** Extracts from P-Scale level descriptors – English.

Level	Language
P1(i)	Pupils encounter activities and experiences. They may show simple reflex responses, *for example, startling at sudden noises or movements.* Any participation is fully prompted’.
P1(ii)	Pupils show emerging awareness of activities and experiences. They may have periods when they appear alert and ready to focus their attention, *for example, attending briefly to interactions with a familiar person.* They may give intermittent reactions.
P2(i)	Pupils begin to respond consistently to familiar people, events and objects. They react to new activities. They begin to show interest in people, events and objects, *for example, smiling at familiar people.* They accept and engage in coactive exploration.
P2(ii)	Pupils begin to be proactive in their interactions. They communicate consistent preferences and affective responses, *for example, reaching out to a favourite person.* They recognize familiar people, events and objects. They perform actions, often by trial and improvement, and they remember learned responses over short periods of time. They cooperate with shared exploration and supported participation.
P3(i)	Pupils begin to communicate intentionally. They seek attention through eye contact, gesture or action. They request events or activities, *for example, pointing to key objects or people.* They participate in shared activities with less support. They sustain concentration for short periods. They explore materials in increasingly complex ways. They observe the results of their own actions with interest. They remember learned responses over more extended periods.
P3(ii)	Pupils use emerging conventional communication. They greet known people and may initiate interactions and activities. They can remember learned responses over increasing periods of time and may anticipate known events. They may respond to options and choices with actions or gestures. They actively explore objects and events for more extended periods. They apply potential solutions systematically to problems.
P4 speaking	Pupils repeat, copy and imitate between 10 and 50 single words, signs or phrases or use a repertoire of objects of reference or symbols. They use single words, signs and symbols for familiar objects and to communicate about events and feelings.
P4 listening	Pupils demonstrate an understanding of at least 50 words, including the names of familiar objects. Pupils respond appropriately to simple requests which contain one keyword, sign or symbol in familiar situations, for example, ‘Get your coat’, ‘Stand up’ or ‘Clap your hands’.

*Source:* Level descriptors P1 to P8 © Qualifications and Curriculum Authority 2009 Great Britain

In addition, the existing data collection forms, which included information related to demographic, medical and need for and availability of assistive devices, were expanded to include additional information.

It is to be noted that the functional items are to be reassessed at regular intervals to monitor change in status. However, the repeated measurements were not tested as part of this pilot.

### Procedure

The process was started in November 2013 with the support of the WCED. Collaboration between members of the CSPID team and the University of Cape Town was initiated. Permission was obtained from the Human Research Ethics Committee of the University of Cape Town (HREC REF: 109/2016). The entire group met and agreed in principle that a database should be developed. Approximately eight meetings were held in the course of the next 12 months including two training sessions on the use of the WeeFIM and the P-scales. A smaller task team was then established to continue this work. An initial list of items was circulated to the larger group, and based on the responses a prototype database was developed and registered with the HREC of the University of Cape Town. The prototype was subjected to a feasibility study with 20 participants. Based on these results, further amendments were made, including the use of drop-down boxes to ensure standardisation of responses under each item.

All participating children had been admitted to the programme prior to the introduction of the database. They had an existing questionnaire, filled in for each child at the specific centre, with relevant data. Additional information was then added to their records. Based on information previously gathered at admission, as well as after the administration of the standardised instruments, a research assistant then collected all this data from the CSPID files, using a survey instrument application, Magpi (http://home.magpi.com/).

Since the inception of the database, consent for including the information of the children enrolled in the CSPID programme and for auditing of procedures has been added to the admission forms. Informed consent was obtained from the parents of all enrolled children. Confidentiality was ensured by removing the names of the children and their centres from the data set prior to analysis.

### Data analysis

Descriptive statistics were used throughout. The three therapists who had utilised the database discussed the statistical outcomes and the value of the information gained from each item until consensus was reached as to the utility and feasibility of each of these headings.

## Results

Of the 134 children recruited, 58% were male. Three quarters of the children spoke Afrikaans, and with three exceptions the others spoke isiXhosa.

**FIGURE 1:**
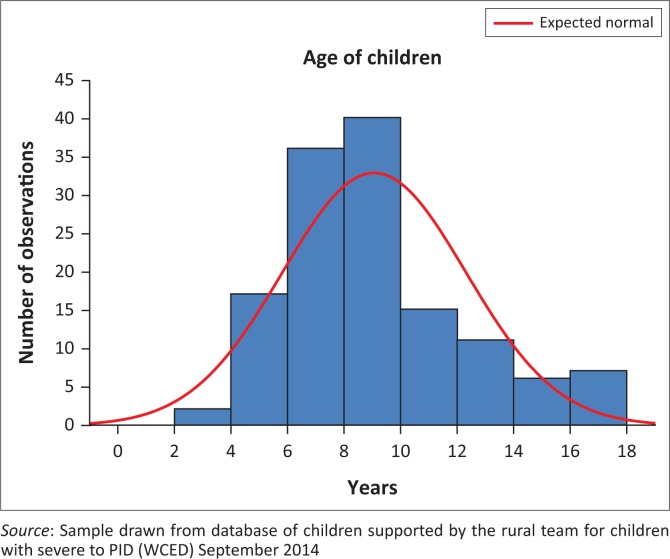
Histogram of the ages of the children (*N* = 134).

Names, surnames and date of birth were 100% available at the time of the study and only one centre did not record home addresses. The mean age was 9.1 years (Standard deviation [SD] = 3.2 years, range 2.7–17.6 years; see Figure 1). Table 5 shows the categories, in terms of grants received, found in the folders. The receipt of grants was unrecorded in 96 of the cases. Other items, such as hospital folder numbers, parental income or socio-economic status were not found to be widely available.

**TABLE 5:** Results on item – grants.

Type of grant	No. of children
Child support	11
Disability	17
Foster	10
Unknown	96

*Source:* Sample drawn from database of children supported by the rural team for children with severe to PID (WCED) September 2014

Data on meeting the need in terms of transport were easily accessible. All children had access to transport arranged or supplied by the SCC.

Where the diagnosis was available, it was entered into the database as written in the folder with cerebral palsy (CP), idiopathic intellectual disability and epilepsy being the most common. It was necessary to recode certain diagnoses (Table 6) for the sake of standardisation; for example, hemiplegia, spastic CP and athetoid were recoded as CP. The results of the items related to the availability of appliances are given in Table 7. Only three children were identified as being in need of an appliance.

**TABLE 6:** Results on item – health conditions.

Variable	Count	Children (%)	Conditions (%)
Cerebral palsy	58	43.3	24.3
Intellectual disability	33	24.6	13.8
Epilepsy	26	19.4	10.9
Chromosomal	16	11.9	6.7
Autism spectrum disorder	13	9.7	5.4
Visual impairment	11	8.2	4.6
Foetal alcohol syndrome	10	7.5	4.2
Microcephaly	10	7.5	4.2
Hydrocephalus	8	6	3.3
Hearing impairment	7	5.2	2.9
Meningitis	6	4.5	2.5
Attention deficit hyperactivity disorder	5	3.7	2.1
Speech impairment	5	3.7	2.1
Muscular-skeletal impairment	5	3.7	2.1
Global developmental delay	4	3	1.7
Kidney/liver disorder	4	3	1.7
Congenital neurological abnormality	3	2.2	1.3
Behaviour disorder	2	1.5	0.8
Spina bifida	2	1.5	0.8
Allergic rhinitis	2	1.5	0.8
Other	6	4.5	2.5
Unknown	3	2.2	1.3

Total	239	-	100

*Source:* Sample drawn from database of children supported by the rural team for children with severe to PID (WCED) September 2014

*N* = 134 children, 239 health conditions.

**TABLE 7:** Results on item – availability of appliances.

Variable	Buggy	Wheelchair	Splint	Standing frame	Hearing aid	Spectacles
No	90	124	127	134	134	132
Yes	39	7	6	0	0	2
Unknown	4	2	6	0	0	0
Need one†	1	1	1	†	†	†

*Source:* Sample drawn from database of children supported by the rural team for children with severe to PID (WCED) September 2014

† The need for visual and hearing aids was not tested.

**FIGURE 2:**
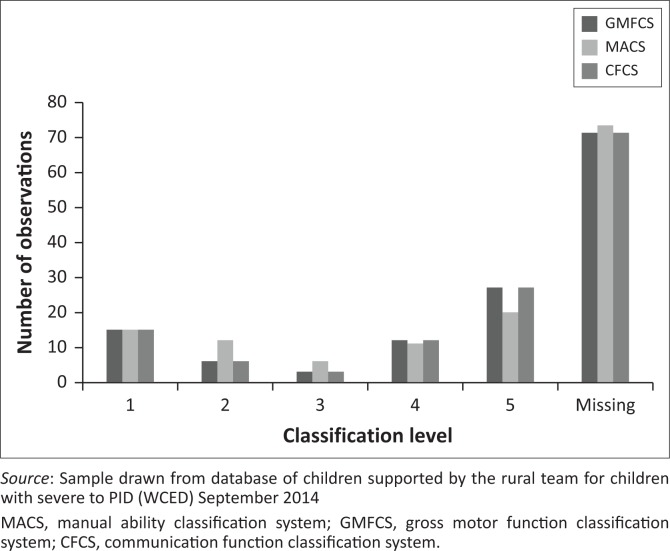
Number of children (y-axis) scoring at the different MACS, GMFCS AND CFCS levels (x-axis).

**FIGURE 3:**
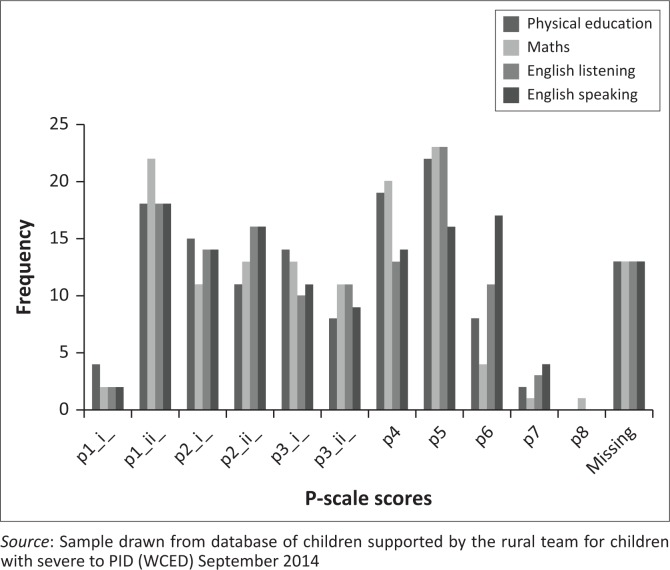
Frequency (y-axis) of P-scale scores (x-axis) across all subject areas.

The classification systems were applied only to those children who had been diagnosed with CP (Figure 2), a sample of 63. Peaks were observed at mild levels (Levels I and II) and unable to do (Level V) for the MACS and GMFCS. In contrast, a steadily increasing percentage of children had problems with communication, with two thirds falling in the most severe category. Similar peaks were observed with the scores on the P-scales, with peaks at p1.ii and p4 and p5 (Figure 3, presentation of P-scale scores of 122 children)

The WeeFIM was administered to 27 children, with the results of the self-care domain recorded in Table 8, as an example of the results obtained. Because of the administrative burden, a small sample of convenience was used.

**TABLE 8:** WeeFIM – self-care domain.

Assistance	Bowel management	Bladder management	Toileting	Dressing lower body	Dressing upper body	Grooming	Eating
1 Total	16	16	17	16	16	13	11
2 Maximal	1	1	2	7	7	8	2
3 Moderate	1	1	-	-	-	-	2
4 Minimal	1	1	-	1	1	-	1
5 Supervision	-	-	3	2	2	5	6
6 Modified independence	1	1	-	-	-	1	-
7 Complete independence	7	7	5	1	1	-	5

**Total**	**27**	**27**	**27**	**27**	**27**	**27**	**27**

*Source:* Sample drawn from database of children supported by the rural team for children with severe to PID (WCED) September 2014

Due to logistical constraints, not all of the 134 children were tested using all of the instruments, resulting in the missing data reported in Figures 2 and 3.

The three therapists involved discussed the feasibility of each item and reached consensus regarding the utility, feasibility and possibility of standardisation thereof, to ensure future reliability. Based on the results of the pilot testing, several items were altered. Items deemed not to meet the criteria of these three constructs were deleted or amended. Their conclusions and suggested amendments are listed below.

The child’s home language was indicated in the centre folder. Apart from Afrikaans and Xhosa as preferred language, the minor exceptions were French, Tsonga or Sotho.

The use of the national identification (ID) number rather than a yet-to-be-allocated CEMIS number is preferred. A unique number is necessary to ensure confidentiality, and it had been thought that a CEMIS number would be the most appropriate. Identity documentation is available in the majority of centres and accompanied by birth certificates, in most cases. Initially, it was thought that hospital numbers could assist with health-related queries. It became apparent that ID numbers would best serve as the reference number in the attempt to align databases from different government departments as well. ID numbers should be utilised as the unique identifying number, which could also be used for children not attending SCCs.

Information with regard to the age of children is extremely important and was available for all. The results identified the need for early intervention and raises questions as to why there was a sudden drop in attendance after the age of 10 years. Another useful finding was that there are several older children who are still attending the centres. Age-related information will be useful both in terms of practical planning of age-appropriate programmes and interventions, as well as for monitoring and planning of future service delivery.

It was agreed that the collection of socio-economic data was difficult to source and unreliable. It was decided to replace employment status with family structure. Similarly, Hospital Payment Scale, which did not render the desired information, was replaced with income category. The Screening Identification Assessment and Support (SIAS) document (Department of Basic Education 2014) acknowledges the essential role of parents in the education and the development of an individual support plan for their child. After data collection, it became apparent that the term disability grant was used incorrectly, as it applies to adults. It was decided to specify the options as follow: *child support*, *care dependency*, *foster care* or *combined foster* and *care dependency grants*. Although there were many missing entries, the team agreed that this was important information which should be gathered in future, as this indicates whether a child receives the appropriate support from the state or not. Should the latter be found, the parent can be guided to the relevant authorities.

The health conditions were available in the folders, but the coding was unstandardised. Intellectual disability was only indicated on a number of folders, mainly those with no other condition. These children with no other diagnosis indicated in their folders are represented in Table 3 as intellectual disability. The team therefore suggested that the International Classification of Disease (World Health Organization 2008) codes be utilised for the primary diagnosis. This diagnosis informs the medical, nursing and therapeutic interventions that might be required. In some cases, there was no clear diagnosis recorded, and the team suggested that there be a second health condition code titled ‘Probable health condition’ which would be based on the clinical symptoms and the judgement of the team members. It is suggested that the third health condition code be related to the aetiology of the condition and that the Global Burden of Disease categories should be utilised. It was agreed that although it might be difficult to gather accurate information, it was important to include the health condition as to inform prevention of conditions, progression of the conditions and management. The coding of the medication was also problematic (although not reported on above) as in some cases, it was coded under generic names, others under brand names and sometimes in terms of the indication for the medication. The final suggestion was that besides coding the name of the medication, the type of medication should be indicated. It was agreed that information relating to chronic medication should be gathered, as it would give an indication of the need for possible nursing support to the SCCs.

With regard to appliances, there was inconsistency between the number of children reported to have spectacles and hearing aids and the number of children reported to have visual or hearing impairments. This highlighted the need for screening of sensory impairments before judging the need of these assistive devices. The absence of an item related to Alternative, Augmented Communication was also noted, and this was added to the list of assistive devices.

The intervention that the child receives from sources other than CSPID should be entered under the headings medical, therapeutic and other stimulation activities, instead of simply medical intervention.

The classification systems, as standardised measures of gross motor, manual and communication ability, has a low administrative burden, is robust and can be used across disciplines. The functional abilities of the children have implications on how they can participate in stimulation activities. Activities need to be adjusted, to accommodate for these functional limitations. The classification systems, thus aid in the easy identification of the children in need of therapeutic intervention to optimise their participation to classroom activities.

Rather than using all the P-Scales, it was suggested that the most useful ‘subject areas’ be identified, renamed and implemented. ‘English’ were substituted with *Language* – *Speaking and Listening* – to cover all other official Languages used in the SCCs. The home language of the child will thus be targeted, and not one specific, preselected Language. ‘Using and Applying Mathematics’ was chosen. These two represented priority areas and *Physical Education* was added to represent an additional skill, according to subject areas outlined in the UK guidelines (Qualifications and Curriculum Authority 2001). These three were chosen, as they correlate to the domains of motor development, cognitive development as well as communication and language development. These were the areas stipulated in the Draft Framework for Therapeutic and Stimulation Programme: Children with Severe to Profound Intellectual Disability (CSPID) developed by the Provincial CSPID team of WCED in November 2012.^3^ Personal and Social Health Education (PSHE) and Citizenship (P-Scales), representing social-emotional skills, formed part of the initial subject areas under investigation, but due to logistical reasons, not implemented to the same extent, and thus not incorporated into the data set. For feasibility of execution in the field, it was unrealistic to incorporate more subject areas. These items appeared to be sufficient to plan basic educational activities and monitor progress at this stage.

The WeeFIM was found to be too time consuming for routine collection and excluded from the final database, although it could be useful at an individual level.

The final version of the database is presented in the Appendix 1.

## Discussion

This paper has documented the development and pilot testing of a database of routinely collected data for CSPID and explored the utility of this data. Based on the results of the pilot testing, several items in the prototype database were altered. There were several lessons learned from the pilot and, as most of the information was relatively easy to access, the therapists expressed confidence in the results. However, where items, such as hospital folder numbers, parental income or socio-economic status, were not found to be available, the utility of having such an item was questioned. It was brought to the attention of the authors that access to data of the Western Cape Department of Health can be gained by the use of identity numbers or name and surname with date of birth. Referrals are made to local clinics or district-based rehabilitation services. Hospital numbers are thus not essential and in effect redundant.

During the course of data collection, it came to the attention that a rare few children had access to private medical care. This elicited a debate about whether it can be assumed that all children in this population had similar socio-economic circumstances or whether this should form part of routine data collection. The learner profile as described in the SIAS document (Department of Basic Education 2014) only includes information on family structure and type of social grant. It is therefore suggested to suffice with these two items, in line with SIAS documentation, as part of routine data collection for CSPID at this stage.

Higgs, NT (Higgs 2007) states that ‘self-reported income data in South Africa is notoriously unreliable’. The inclusion of income category should therefore be reconsidered. In the Support Needs Assessment part of the SIAS document (Department of Basic Education 2014) the following other factors are mentioned as possible barriers to learning, namely: number of schools attended, refugee/immigrant status, substance abuse, domestic violence, divorce, neglect, disabled or ill parents and poverty stricken background. Models, like the American Association on Intellectual and Development Disabilities conceptual framework for human functioning (Buntinx & Schalock 2010), propose an outline to integrate activities from different disciplines involved in service delivery to people with intellectual disability. Context forms a crucial part of measuring Human Functioning, as proposed by Buntinx and Schalock. It is advised by the authors that role players draw from items suggested by Higgs (2007) and Berry *et al*. (2013). Part three of South African Child Gauge 2013 also outlines vital pointers on the socio-economic rights of children, which are a subset selected from the website www.childrencount.ci.org.za. As the above mentioned factors have a high prevalence in people with intellectual disabilities (Adnams 2010), future inclusion of these items might have to be considered by the relevant authorities.

For other items, such as health condition and medication, the information was generally available although challenges remain. By default, all of the children admitted to a SCC are because of intellectual disability and it should therefore be excluded from the list of conditions. It was also considered necessary to provide more details regarding the interventions that the children received.

It became clear on analysis that such a database will collect much useful information that can inform the nature, content and extent of service delivery to CSPID and monitor the impact of such service. For instance, the pilot revealed that there were very few children under the age of 4 years attending the centres. The percentage reflected in this study is lower than the 25% of CDG beneficiaries between 0–6 years of age, found to be attending early learning facilities in the local community (Department of Social Development, DWCPD & UNICEF 2012). This raises the question as to whether young CSPID are receiving early intervention, and if so, to what extent and of what nature? It is beyond the scope of this paper to really contemplate the reason for the sudden drop in numbers at the age of 10. However, appropriate school placement (Department of Social Development *et al*. 2012) and severe behavioural challenges (Carvill 2001) come to mind, but there will surely be other factors to consider. The number of older children who will be leaving the centres speaks to the need to have appropriate care centres for young adults, as well as improving the education of both younger children and adolescents. As so many children presented with health conditions, which would influence physical functioning and/or require medication, it is clear that a multidisciplinary team, including educationalists and allied health professionals such as therapists, is essential. The small numbers of children still in need of physical assistive devices indicates that delivery of appliances, such as wheelchairs and buggies, has been appropriate and successful. On the other hand, very few children with sensory impairments received aids such as spectacles, hearing aids and communication devices. This highlights the need for adequate screening of sensory impairments. Given the high prevalence of sensory impairments (Department of Social Development *et al*. 2012) and the resultant impact on learning, this should be prioritised.

The language used by staff members in the centres did not get reflected in the original data set. The language of learning and teaching is important in terms of planning service delivery. The data on home language might appear to be in contrast to the figures for the whole of the WC, but is the reality for the rural parts of the province, which were included in the study. Only one of the centres used Xhosa as the language of instruction, one centre used both Afrikaans and English and the others instructed mainly in Afrikaans. As the focus of the team is on communication and not teaching a specific language, using the home language of the child is encouraged as far as possible.

Availability of transport is of utmost importance. Most children cannot afford personal or public transport, which would result in non-attendance or a high degree of absenteeism. Unfortunately, this database underestimates the need for transport as it does not reflect the number of children not gaining access to the SCC because they reside outside the area covered by centre transport. The need in terms of transport, in reality, is thus bigger than reflected in these statistics.

For many children the reason for not attending a SCC is probably or partly due to inaccessibility of transport. The need is to expand the use of the data set for children on a waiting list at that particular centre. In doing so, it will be a start to account for children whose rights have not been met. It is beyond the logistical constraints of the multifunctional teams to venture further out into the community. In the metro areas, a non-profitable organisation (NPO) is employed by the Department of Social Development to develop home programs for children with no access to SCC’s or schools. In the rural areas, due to the vast distances involved, the authors are of the opinion that if would be more feasible to strengthen the means of the rural CSPID team, with the brief to support out of centre children as well.

The need for therapeutic expertise, across the disciplines, is indicated by the large numbers of children with CP, classified at the most severe levels of the MACS, GMFCS and the CFCS. Although initially developed specifically for use in CP (Bodkin, Robinson & Perales 2003), there is a need to utilise these systems with children with other diagnoses apart from CP. Although the items appear to be sufficiently generic to apply to children with other diagnoses, the validity of this use will need to be established. The Gross Motor Function Classification System (GMFCS), although robust and suitable to describe how severe the child with CP is affected, it was not developed with the purpose of measuring difference over time or subsequent to intervention (Adams 2009). The same is true for the other classification systems. Each discipline will thus have to further investigate appropriate outcome measures within their respective scope of service delivery. This is a fragmentary approach, probable only possible to administer on a carefully selected few. Although the Vineland Screener 0-12 years research version (Van Duijn *et al*. 2009) was discarded as a measurement for routine use, it is suggested that Vineland Adaptive Behavior Scales (2^nd^ ed.) (Sparrow, Cicchetti & Balla 2005) be reconsidered. It is standardised and has a more holistic approach which includes most of the domains applicable to CSPID.

The Vineland Adaptive Behavior Scales (2^nd^ ed.) (Sparrow, Cicchetti & Balla 2005) assesses adaptive behaviour, which determines severity and consequently the level of support required (American Psychiatric Association 2013). The DSM-5 defines the conceptual domain for PID as the following:

Conceptual skills generally involve the physical world rather than symbolic processes. The individual may use objects in goal-directed fashion for self-care, work, and recreation. Certain visuospatial skills, such as matching and sorting based on physical characteristics, may be acquired. However, co-occurring motor and sensory impairments may prevent functional use of objects.

The P-Scales were found to be very useful not only as a measure of outcome but also to assist in planning the educational support and developing appropriate activities and can also support the implementation of a curriculum. It serves as a common tool shared by educators and health-care professionals. It became clear that this was the assessment tool of choice with which to measure educational progress, particularly as elements thereof will probably be integrated into the new national curriculum for children with intellectual impairments still being developed, by a task team for the National Department of Education.^4^

It is a low-cost high-relevance tool which can be implemented in the interim alongside the South African National Curriculum Framework (NCF) for children 0–4 years (Department of Basic Education 2014). The content of this curriculum addresses the cognition of children 0–4 years and can partly address the learning needs of CSPIDs of all ages. P-Scales as an assessment tool can be implemented from the age of 5 years. Yet the principles of exposure to stimulation, coactive exploration and supported participation can be applied to all, irrespective of age.

To align with the subject areas outlined in the NCF, the terms *Communication* and *Exploring Mathematics* are suggested until the final PID curriculum becomes available. It is imperative that the subject of PSHE and Citizenship become part of the base line data set. It is advised that thought should be given to the eventual inclusion of *Music* as well as *Art and Design* as subject areas, addressing the priority area of sensory awareness and perception (Qualifications and Curriculum Authority 2001). Given the contextual constraints and the fact that the project is still being developed, the authors are of the opinion that the current inclusion of more subject areas is not currently advisable.

The distribution of peaks, which indicates a substantial number of children are performing at a relatively high level, may be useful in identifying children who may be performing at higher levels and are thus inappropriately placed in centres for CSPID. At an individual level, the results of the P-Scales can be utilised to target specific areas of weakness. As case discussions with parents and care staff are part of routine work for CSPID, it will be possible to integrate P-Scales into this process. These guidelines thus give all those involved in the educational stimulation of the child a ‘small steps framework’ (Mittler 2002) that responds to the diverse educational needs of CSPID. It is a very practical guide to appropriately address learning challenges, which respond to diverse need in learning, facilitate inclusion and display ways to overcome barriers to learning.

Regular, routine P-Scale assessments will enable charting of the learners’ progress. This information is useful to monitor progress over time, assess the effectiveness of intervention and to identify where further support is needed. At a policy level, an analysis of the differential performance on the different scales can inform curriculum development and lead to a greater understanding of the inter-relationship of the different skill categories (Qualifications and Curriculum Authority 2001). In addition, those centres or intervention programmes that are associated with the greatest improvement in the learners’ functioning should be identified and should be emulated. The authors recommend that the P-Scale results to be administered by CEMIS, just as the examination results of mainstream school children are retained and coordinated. In this way, each child will become visible to the relevant authorities.

However, the appropriate use of the scales requires training to ensure standardisation of assessment across children, team members and centres. This training should be incorporated into capacity building and professional development of CSPID team members.

The use of the WeeFIM is not recommended for the general routine assessment of CSPID. Although the WeeFIM is a standardised assessment tool, it includes the term of maximal independence. Children with profound disability will always be dependent in many areas of self-care and other activities of daily life. With the exclusion of the WeeFIM due to cost and time implications, the database had a gap in the important area of self-care assessment. The authors identified the need to develop or identify an appropriate self-care classification system for children comparative to the other classification systems. This is a matter of urgency, as developing self-care skills is an essential component in educating CSPID. It is advisable that this tool be developed within the contemporary framework of a support model (Buntinx & Schalock 2010) which takes into account not only the child’s abilities but contextual specific elements.

The development of the database was supported by grant funding, and the collection and entry of the data onto a mobile tablet were done by a research assistant, supported by the grant. The analysis and reporting were done in collaboration with academic partners at the University of Cape Town. The process was enriching for both parties and demonstrated the value of academic and public partnerships. However, this model is not sustainable and there is a need to institutionalise the collection, storage, analysis and dissemination of reports and monitoring of the results. This would, in the light of the ruling of the High Court, appear to be a responsibility of the education authorities who have the capacity and structure within CEMIS to perform these functions. Hopefully, this initiative of the CSPID team in identifying and piloting a viable database will inform the future integration of information relating to CSPID into the system that collects and manages equivalent data for all children attending schools. This will go some way in ensuring that the educational and other needs of learners who have severe and profound cognitive impairments are adequately addressed, especially those that have not been enrolled in centres or accommodated in schools. In time, after implementation in the field, the resultant core data set has the potential to form a reference to other countries in Southern Africa, possibly even to more low- to middle-income countries.

## Conclusions

It is clear that the development of this database represents the first steps on a long road. In the near future, the use of the database can be rolled out in all the CSPID districts, and repeated assessments of functioning at regular intervals can start. There is no doubt that the database will undergo many amendments as deficiencies and redundancies are identified with use. However, it is likely that a core set of information will be collected and over time, an overall picture of the needs and capabilities of CSPID will emerge. This information will be useful for service planning and monitoring. At an individual level, regular assessment of the performance levels of the children will give parents, therapists and care workers useful information not only with regard to current performance, strengths and challenges but also in terms of the development and emerging needs of the children. The authors maintain that if a system of data collection is instated by the educational authorities, these most vulnerable of children will become visible and their right to education, as endorsed by the court ruling, will be adequately addressed.

## Footnotes

1 This process is described in a paper by McKenzie J, Pillay N, Duvenhage CM, Du Plessis E and Jelsma J: ‘Implementation of educational provision for children with severe and profound intellectual disability in the Western Cape: From rights to reality which is in preparation’.

2 Report on Activities of the Provincial Teams for Children with Severe to Profound Intellectual Disabilities (CSPID), for the period April 2012 to March 2013, submitted to the WCED.

3 Draft Framework for Therapeutic and Stimulation Programme: Children with severe to profound intellectual disability, developed by Provincial CSPID team November 2012, submitted to WCED.

4 Department of Basic Education (2015) The draft South African policy framework for the provision of quality education and support to children and youth with profound intellectual disability.

## Appendix 1

Appendix: Items included in the final database

1. Unique number
2. Surname
3. Name
4. Date of birth
5. Address
6. Centre
7. CSPID team linked to centre
8. Home language
9. Gender
10. Social circumstances home – family structure
11. Social economic status – family income category
12. Grant
13. Transport
14. Assistive devices – wheelchair
15. Buggy
16. Splints
17. Standing frame
18. Hearing aid
19. Glasses
20. AAC
21. Level of educational support
22. ICD11 diagnosis
23. Probable health condition
24. Co-morbid probable health condition
25. Aetiology
26. Chronic medical conditions
27. Outcome – what has happened to the child who left the centre
28. Chronic medication category
29. Chronic medication 1
30. Chronic medication 2
31. Medical intervention
32. Therapeutic intervention (non-CSPID)
33. Other services available at the centre, for example, horse riding
34. Date of assessment of classification systems (GMFCS, MACS and CFCS) (self-care)
35. MACS score
36. GMFCS score
37. CFCS score
38. Self-care classification score – feeding
39. Self-care classification score – drinking
40. Self-care classification score – dressing
41. Self-care classification score – tooth brushing
42. Self-care classification score – toileting
43. Self-care classification score – total
44. Date of assessment P-scales
45. P-Scale score Communication – speaking
46. P-Scale score Communication – listening
47. P-Scale score Exploring Mathematics
48. P-Scale score Physical Education
49. CSPID interventions
50. Date of reassessment of classification systems
51. MACS score
52. GMFCS score
53. CFCS score
54. Self-care classification score – feeding
55. Self-care classification score – drinking
56. Self-care classification score – dressing
57. Self-care classification score – tooth brushing
58. Self-care classification score – toileting
59. Self-care classification score – total
60. Date of reassessment P-Scales
61. P-Scale score Communication – speaking
62. P-Scale score Communication – listening
63. P-Scale score Exploring Mathematics
64. P-Scale score Physical Education

## References

[CIT0001] Adams J.V., 2009, ‘Understanding function and other outcomes in cerebral palsy’, *Physical Medicine and Rehabilitation Clinics of North America* 20, 567–576. http://dx.doi.org/10.1016/j.pmr.2009.04.002 19643354 10.1016/j.pmr.2009.04.002 PMC2719719

[CIT0002] Adnams C.M., 2010, ‘Perspectives of intellectual disability in South Africa: Epidemiology, policy, services for children and adults’, *Current Opinion in Psychiatry* 23, 436–440. http://dx.doi.org/10.1097/YCO.0b013e32833cfc2d 20683179 10.1097/YCO.0b013e32833cfc2d

[CIT0003] American Psychiatric Association, 2013, *Diagnostic and statistical manual of mental disorders*, 5th edn., American Psychiatric Association, Arlington, Washington, DC.

[CIT0004] Berry L., Biersteker L., Dawes A., Lake L. & Smith C., 2013, *South African child gauge 2013*, Children’s Institute, University of Cape Town, Cape Town.

[CIT0005] Bodkin A.W., Robinson C. & Perales F.P., 2003, ‘Reliability and validity of the gross motor function classification system for cerebral palsy’, *Paediatric Physical Therapy* 15(4), 247–252. http://dx.doi.org/10.1097/01.PEP.0000096384.19136.02 10.1097/01.PEP.0000096384.19136.02 17057460

[CIT0006] Buntinx W.H.E. & Schalock R.L., 2010, ‘Models of disability, quality of life, and individualized supports: Implications for professional practice in intellectual disability’, *Journal of Policy and Practice in Intellectual Disability* 7, 283–295. http://dx.doi.org/10.1111/j.1741-1130.2010.00278.x

[CIT0007] Carvill S., 2001, ‘Sensory impairments, intellectual disability and psychiatry’, *Journal of Intellectual Disability Research* 45, 467–483. http://dx.doi.org/10.1046/j.1365-2788.2001.00366.x 11737534 10.1046/j.1365-2788.2001.00366.x

[CIT0008] Department for Education, 2014, *Performance – P Scale – Attainment targets for pupils with special educational needs*, viewed 18 June 2015, from https://www.gov.uk/government/uploads/system/uploads/attachment_data/file/329911/Performance_-_P_Scale_-_attainment_targets_for_pupils_with_special_educational_needs.pdf

[CIT0009] Department of Basic Education, 2014, *Policy on screening identification assessment and support*, Department of Basic Education, Pretoria.

[CIT0010] Department of Basic Education, 2015, *The South African National Curriculum Framework for children from birth to four*, Department of Basic Education, Pretoria.

[CIT0011] Department of Social Development, DWCPD & UNICEF, 2012, *Children with disabilities in South Africa: A situation analysis 2001–2011*. Department of Social Development/Department of Women, Children and People with Disabilities/UNICEF, Pretoria.

[CIT0012] Eliasson A.C., Krumlinde-Sundholm L., Rosblad B., Beckung E., Arner M., Ohrvall A.M., et al, 2006, ‘The manual ability classification system (MACS) for children with cerebral palsy: Scale development and evidence of validity and reliability’, *Developmental Medicine and Child Neurology* 48, 549–554. http://dx.doi.org/10.1017/S0012162206001162 16780622 10.1017/S0012162206001162

[CIT0013] Hidecker M.J., Paneth N., Rosenbaum P.L., Kent R.D., Lillie J., Eulenberg J.B., et al, 2011, ‘Developing and validating the communication function classification system for individuals with cerebral palsy’, *Developmental Medicine and Child Neurology* 53, 704–710. http://dx.doi.org/10.1111/j.1469-8749.2011.03996.x 21707596 10.1111/j.1469-8749.2011.03996.x PMC3130799

[CIT0014] Higgs N.T., 2007, ‘Measuring and understanding the well-being of South Africans: Everyday quality of life in South Africa’, *Social Indicators Research* 81, 331–356. http://dx.doi.org/10.1007/s11205-006-9012-3

[CIT0015] Kleintjies S., Flisher A.J., Fick M., Railoun A., Lund C., Molteno C., et al, 2006, ‘The prevalence of mental disorders among children, adolescents and adults in the western Cape, South Africa’, *South African Psychiatry Review* 9, 157–160. http://dx.doi.org/10.4314/ajpsy.v9i3.30217

[CIT0016] McDowell B., 2008, ‘The gross motor function classification system – Expanded and revised’, *Developmental Medicine and Child Neurology* 50, 725 http://dx.doi.org/10.1111/j.1469-8749.2008.03104.x 18834382 10.1111/j.1469-8749.2008.03104.x

[CIT0017] McKenzie J., McConkey R. & Adnams C., 2013a, ‘Health conditions and support needs of persons living in residential facilities for adults with intellectual disability in the Western Cape Province’, *South African Medical Journal* 103(7), 481–484. http://dx.doi.org/10.7196/SAMJ.6491 23802215 10.7196/samj.6491

[CIT0018] McKenzie J.A., McConkey R. & Adnams C., 2013b, ‘Intellectual disability in Africa: Implications for research and service development’, *Disability and Rehabilitation* 35, 1750–1756. http://dx.doi.org/10.3109/09638288.2012.751461 23350758 10.3109/09638288.2012.751461

[CIT0019] Milne S., McDonald J. & Comino E.J., 2012, ‘The use of the Bayley scales of infant and toddler development III with clinical populations: A preliminary exploration’, *Physical & Occupational Therapy in Pediatrics* 32, 24–33. http://dx.doi.org/10.3109/01942638.2011.592572 21812743 10.3109/01942638.2011.592572

[CIT0020] Mittler P., 2002, ‘Educating pupils with intellectual disabilities in England: Thirty years on’, *International Journal of Disability, Development and Education* 49, 145–160. http://dx.doi.org/10.1080/103491220141730

[CIT0021] Ottenbacher K.J., Msall M.E., Lyon N., Duffy L.C., Ziviani J., Granger C.V., et al, 2000, ‘The WeeFIM instrument: Its utility in detecting change in children with developmental disabilities’, *Archives of Physical Medicine and Rehabilitation* 81, 1317–1326. http://dx.doi.org/10.1053/apmr.2000.9387 11030496 10.1053/apmr.2000.9387

[CIT0022] Piper M.C., Pinnell L.E., Darrah J., Maguire T. & Byrne P.J., 1992, ‘Construction and validation of the Alberta Infant Motor Scale (AIMS)’, *Canadian Journal of Public Health* 83(Suppl 2), S46–S50. 1468050

[CIT0023] Qualifications and Curriculum Authority, 2001, *Developing skills. Planning, teaching and assessing the curriculum for pupils with learning difficulties*, Qualifications and Curriculum Authority, London.

[CIT0024] Qualifications and Curriculum Authority, 2009, *The P scales. Level descriptors P1–P8*, Qualifications and Curriculum Authority, London.

[CIT0025] Robson C. & Evans P., 2003, *Educating children with disability in developing countries: The role of data sets*. The University Repository, The University of Huddersfield, Huddersfield, UK.

[CIT0026] South African Legal Information Institute, 2007, *Western Cape Forum for Intellectual Disability v. Government of the Republic of South Africa & Government of the Province of Western Cape*, Case no: 18678/2007, viewed 18 June 2015, from http://www.saflii.org/za/cases/ZAWCHC/2010/544.html

[CIT0027] Sparrow S.S., Cicchetti D.V. & Balla D.A., 2005, ‘Vineland Adaptive Behavior Scales (2nd ed.)’ *American Guidance Service*

[CIT0028] Tafiadis D., Paroutiadou I., Papageorgiou K. & Tafiadis M., 2010, ‘The expressive and the receptive one word picture vocabulary test (EOWPTT & ROWPVT). (A combine pilot study and validation of the tests’ in normal Greek population – Aged from 11 years till 11 years and 11 months)’, *Annals of General Psychiatry* 9, 1. 10.1186/1744-859X-9-S1-S117 PMC2991800 25396452

[CIT0029] United Nations, 2015, *United Nations Millennium goals*, viewed 18 June 2015 from http://www.un.org/millenniumgoals/

[CIT0030] Van Duijn G., Dijkxhoorn Y., Noens I., Scholte E. & Van Berckelaer-Onnes I., 2009, ‘Vineland Screener 0–12 years research version (NL). Constructing a screening instrument to assess adaptive behaviour’, *International Journal of Methods in Psychiatric Research* 18(2), 110–117. http://dx.doi.org/10.1002/mpr.282 19507164 10.1002/mpr.282 PMC6878323

[CIT0031] World Health Organization, 2008, *The global burden of disease: 2004 update*, WHO, Geneva, Switzerland.

